# 21-Gene Recurrence Assay Associated With Favorable Metabolic Profiles in HR-Positive, HER2-Negative Early-Stage Breast Cancer Patients

**DOI:** 10.3389/fendo.2021.725161

**Published:** 2021-08-11

**Authors:** Yifei Zhu, Tiange Wang, Yiwei Tong, Xiaosong Chen, Kunwei Shen

**Affiliations:** ^1^Department of General Surgery, Comprehensive Breast Health Center, Ruijin Hospital, Shanghai Jiao Tong University School of Medicine, Shanghai, China; ^2^Shanghai National Clinical Research Center for Metabolic Diseases, Shanghai Institute of Endocrine and Metabolic Diseases, Ruijin Hospital, Shanghai Jiao Tong University School of Medicine, Shanghai, China

**Keywords:** 21-gene recurrence score assay, cancer-related genes, metabolic profile, breast cancer, Chinese women

## Abstract

**Background:**

Comprehensive investigations of the associations between 21-gene recurrence assay and metabolic profiles in Chinese breast cancer patients are limited.

**Methods:**

We evaluated the relations of the 21-gene recurrence risk score (RS) and the expression of cancer-related genes with metabolic factors and biomarkers of insulin and the insulin-like growth factor (IGF) axis, and examined the interactions between the 21-gene RS and these metabolic profiles on breast cancer recurrence in Chinese women with HR-positive, HER2-negative early-stage breast cancer.

**Results:**

The 21-gene RS was inversely associated with body mass index ([BMI]β: −0.178 kg/m^2^; P=0.040), the homeostasis model assessment of insulin resistance index ([HOMA-IR] β: −0.031; P=0.042), insulin (β: −0.036 uIU/ml; P=0.009), and C-peptide (β: −0.021 ug/L; P=0.014) and was positively associated with high-density lipoprotein cholesterol (β: 0.025 mmol/L; P=0.004), which were driven by the relation patterns between specific cancer-related genes and these metabolic profiles. Each 10-unit increase in the 21-gene RS was associated with 28% (95% CI: 5–47%) higher risk of breast cancer recurrence; this association was also observed in patients with favorable metabolic profiles in relevant to an absence of obesity, insulin resistance, hyperglycemia, hypertension, or dyslipidemia (28–44% higher risk) and among women with a low level of insulin, C-peptide, or the IGF1/IGFBP3 ratio (41–155% higher risk).

**Conclusions:**

The 21-gene RS was related to favorable metabolic profiles including lower BMI, HOMA-IR, insulin, and C-peptide, and higher HDL in Chinese breast cancer patients, and its prognostic impact on breast cancer recurrence was more likely to present among patients with relatively favorable metabolic profiles.

## Introduction

Breast cancer is the leading cause of cancer death in women and has now surpassed lung cancer as the most commonly diagnosed cancer (approximately 11.7% of total new cancer cases) worldwide in 2020, according to statistics released by the International Agency for Research on Cancer (1). Estrogen receptor (ER)-positive, human epidermal growth factor-2 (HER2)-negative breast cancer represents the largest subgroup of breast cancer and associates a relatively better prognosis because of its sensitivity to targeted hormonal therapy (2). The 21-gene recurrence assay is a genetic test that applies reverse transcriptase-polymerase chain reaction to examine the expression of 21 genes, including 16 cancer-related genes and five endogenous reference genes, in formalin-fixed paraffin-embedded breast tumors (3, 4). The 21-gene recurrence risk score (RS) is computed on the basis of the expression levels of these genes and has been widely used to predict the risk of distant recurrence and the potential benefit of adjuvant chemotherapy in women with ER-positive, HER2-negative early-stage breast cancer (3–5).

In addition to the 21-gene RS assay, the complex metabolic profiles are closely related to the recurrence of breast cancer. Several metabolic risk factors, such as insulin resistance, hyperglycemia, and dyslipidemia, systemically occur in the setting of adipose inflammation and operate in concert with local mechanisms to maintain an inflamed microenvironment and promote adverse breast cancer prognosis (6–8). Besides, elevated insulin and glucose levels have been observed in patients with early-stage breast cancer exhibiting white adipose tissue inflammation (9). Insulin can stimulate the synthesis of insulin-like growth factor (IGF), and both have potent mitogenic effects on tumor growth (10–13). Therefore, taking into account the 21-gene RS assay, as well as metabolic risk factors and circulating biomarkers, would help accurately characterize the metabolic status of breast cancer patients and identify individuals at increased risk for breast cancer recurrence. Thus far, such investigations are scarce.

In an effort to address this knowledge gap, we aimed to study the associations of the 21-gene RS with metabolic risk factors and circulating biomarkers of insulin and the IGF axis, and characterize the prognosis prediction interaction between the 21-gene RS and metabolic profiles in Chinese women with hormone receptor (HR)-positive, HER2-negative early-stage breast cancer.

## Methods

### Study Setting and Patients

This study included 1,038 patients who were enrolled from the Comprehensive Breast Health Center, Ruijin Hospital, Shanghai Jiao Tong University School of Medicine, between 2012 and 2017. Patients who met the inclusion criteria were included in the analysis: (1) female gender; (2) ER and/or PR-positive, HER2-negative invasive breast cancer; (3) tumor size T1-T3; (4) axillary lymph node involvement N0-N1; and (5) with complete data available on baseline demographic and clinical characteristics, metabolic factors, biomarkers of insulin and the IGF axis, and the 21-gene RS assay. The exclusion criteria were as follows: (1) pT4 or pN2-3 breast cancer; (2) metastatic breast cancer; or (3) previous neoadjuvant systemic therapy. Pathology diagnoses were given from the experienced pathologists by evaluating stored hematoxylin and eosin–stained sections from all patients in the Department of Pathology, Ruijin Hospital. Breast cancer was diagnosed by histopathologic examination according to the fourth edition of the World Health Organization Classification of Tumors of the Breast (14). The study protocol was approved by the Medical Ethics Committee of Ruijin Hospital, Shanghai Jiao Tong University. All study patients provided the written informed consent.

### Clinical Data Collection

Standardized clinical data of all patients enrolled in this study were obtained from Shanghai Jiao Tong University Breast Cancer Database. Baseline demographic and clinical characteristics included age, menopause status, histological grade, TNM stage, tumor size, lymph node status, ER percentage, progesterone receptor (PR) status, Ki-67 level, molecular subtype, and adjuvant therapies. Histological grade, TNM stage, tumor size, and lymph node status were defined according to American Joint Committee on Cancer (AJCC) stage of disease classification (Eighth Edition) (15). ER and PR-positive status was defined as nuclear staining of the tumor cell ≥1%. HER2-negative status was defined as 0 to 1+ by immunohistochemistry or negative of HER2 amplified by fluorescence *in situ* hybridization. Ki-67 index was characterized as the proportion of positively nuclear staining cells among at least 1,000 tumor cells in the area counted (16). Molecular subtypes were classified as luminal A-like subtype (defined as ER-positive, HER2-negative with PR ≥20% and Ki67 <14%) and luminal B-like subtype (defined as ER-positive, HER2-negative with PR <20%, or Ki67 ≥14%) according to the 2013 St. Gallen International Expert Consensus (17). Adjuvant therapies including chemotherapy, radiotherapy, and endocrine therapy were determined by multidisciplinary team discussion.

### Definition and Measurement of Metabolic Profiles

Metabolic risk factors included obesity, insulin resistance, hyperglycemia, hypertension, and dyslipidemia. The biomarkers of insulin and the IGF axis included insulin, C-peptide, IGF1, and IGF-binding protein 3 (IGFBP3). Weight was measured to the nearest 0.1 kg wearing light indoor clothing, and height was measured to the nearest 0.1 cm without shoes. BMI was calculated as weight in kilograms divided by height in meters squared. Obesity was defined as body mass index (BMI) ≥25 kg/m^2^. The homeostasis model assessment of insulin resistance (HOMA-IR) index was calculated as fasting insulin (μIU/ml) × fasting glucose (mmol/L)/22.5. Insulin resistance was defined as HOMA-IR ≥2.5. Hyperglycemia was defined as fasting glucose ≥5.6 mmol/L. Hypertension measured by blood pressure cuff was defined as systolic blood pressure (SBP) ≥140 mmHg or diastolic blood pressure (DBP) ≥90 mmHg. Dyslipidemia was defined as total cholesterol (TC) ≥6.22 mmol/L, high-density lipoprotein (HDL) <1.04 mmol/L, low-density lipoprotein (LDL) ≥4.14 mmol/L, or triglycerides ≥2.26 mmol/L.

Measurements of all metabolic profiles mentioned above were presented in details in our previous work (18, 19). After an overnight fast of at least 8 h, blood samples were obtained before the surgical procedure. Fasting glucose and lipid profiles including TC, HDL, LDL, and triglycerides were collected using Beckman Coulter-AU 5800 (Beckman Coulter, Inc., Atlanta, GA, USA). Serum insulin and C-peptide were measured by electrochemiluminescence immunoassay on Cobas E601 analyzers (Hoffman-La Roche Ltd., Basel, Switzerland). Plasma IGF1 and IGFBP3 were measured by chemiluminescent immunoassay using IMMULITE 2,000 system (Siemens AG, Munich, Germany). The IGF1/IGFBP3 ratio was calculated. All biomarkers were examined at the Clinical Laboratory of Ruijin Hospital.

### Analytic Methods of the 21-Gene RS

The 21-gene RS assay was performed in the Department of Clinical Laboratory, Ruijin Hospital. Detailed information about the 21-gene RS assay has been reported previously (3). Hematoxylin and eosin–stained slides were reviewed to ensure sufficient invasive breast cancer by pathologist in the Department of Pathology, Ruijin Hospital. Then RNA was extracted from three 10 μm unstained sections of formalin-fixed, paraffin-embedded (FFPE) tissue using the RNeasy FFPE RNA kit (Qiagen, 73504, Germany). Gene-specific reverse transcription was performed using an Omniscript RT kit (Qiagen, 205111, Germany). Standardized quantitative reverse transcriptase polymerase chain reaction (RT-PCR) reactions were performed using Premix Ex TaqTM (TaKaRa Bio, RR390A) in an Applied Biosystems 7500 Real-Time PCR System (Foster City, CA, USA). The expression of 16 cancer-related gene was measured in triplicate and normalized relative to a set of five reference genes. The relative expression level of each target gene, in the form of ΔCt value, was defined as Ct gene - Ct reference. The Characteristics of the 21 individual genes are presented in **Supplementary Table 1**. The RS was derived from the reference-normalized expression measurements for the 16 cancer-related genes, ranging from 0 to 100, with a higher score indicating a higher probability of distant recurrence and more benefit gained from chemotherapy. RS were classified as low-risk (RS <18), intermediate-risk (RS 18–30), and high-risk (RS ≥31) categories according to cutoffs used in the test report (3, 20, 21).

### Outcomes

The outcome was breast cancer recurrence, defined as invasive ipsilateral breast tumor recurrence, local/regional invasive recurrence, distant recurrence, ipsilateral ductal carcinoma *in situ* (DCIS), and contralateral DCIS (22).

### Statistical Analysis

Baseline characteristics of study participants by the 21-gene RS were presented as mean (standard deviation, SD) for continuous variables or number (proportion) for categorical variables. Differences between baseline characteristics within low, intermediate, and high categories of the 21-gene RS were compared by general linear model for continuous variables or Chi-square test for categorical variables.

General linear models were applied to examine the associations of the ΔCt value for cancer-related genes and the 21-gene RS with metabolic factors including BMI, HOMA-IR, fasting glucose, SBP, DBP, total cholesterol, HDL, LDL, and triglycerides, as well as the biomarkers of insulin and the IGF axis with, with adjustment for age and postmenopausal status. Levels of HOMA-IR, IGF1, IGFBP3, IGF1/IGFBP3 ratio, insulin, and C-peptide were log transformed in analysis to improve the normality of their distributions.

In the time-to-event analysis for breast cancer recurrence, participants were censored at the date of recurrence or the end of follow-up, whichever occurred first. Person-time was calculated from the enrollment date to the censoring date for each participant. Cox proportional hazards models were used to calculate hazard ratios (HRs) and 95% confidence intervals (CIs) for breast cancer recurrence associated with each 10-unit increase in the 21-gene RS, according to status of obesity, insulin resistance, hyperglycemia, hypertension, and dyslipidemia, as well as levels of insulin, C-peptide, IGF1, IGFBP3, and IGF1/IGFBP3 ratio. Models were adjusted for age, BMI, postmenopausal status, tumor size, lymph node status, PR status, Ki-67 level, molecular subtype, chemotherapy, radiotherapy, and endocrine therapy. Interactions of the 21-gene RS with the stratifications of metabolic risk factors and biomarkers on the risk of recurrence were assessed by including the multiplicative interaction product term (e.g., the RS × obesity status), as well as the main association in the models. All reported P values are nominal and 2-sided, and a P value of less than 0.05 was considered statistically significant. All statistical analyses were performed by using SAS software, version 9.2 (SAS Institute Inc).

## Results

### Baseline Characteristics

Baseline characteristics of 1,038 women with ER-positive, HER2-negative early-stage breast cancer are shown in **Table 1**. The mean values (SDs) of the 21-gene RS were 12.6 (4.3), 24.4 (3.7), and 41.0 (13.2) for the low, intermediate, and high categories of the 21-gene RS, respectively. Compared with participants with a 21-gene RS <18, participants with a higher RS were younger, had higher levels of HDL and IGF1, and had lower levels of insulin and C-peptide. Participants with a higher RS had higher proportions of histological grade III, lymph node-positive, high Ki-67 (≥14), and Luminal B subtype, and lower proportions of high ER% (≥50%) and PR-positive. For adjuvant therapy, participants with a higher RS had higher proportions of chemotherapy and radiotherapy than participants with a 21-gene RS <18.

**Table 1:** Baseline characteristics of breast cancer patients by the 21-gene RS categories^a^.

Characteristic	21-gene RS^b^			P value
	Low (RS <18)	Intermediate (RS 18-30)	High (RS ≥31)
Number of patients	252	572	214	–
Age, year	60.2 (12.6)	56.2 (12.6)	56.9 (11.7)	0.002
Postmenopausal status, n (%)	174 (69.1)	358 (62.6)	151 (70.6)	0.051
*Metabolic factor*				
BMI, kg/m^2^	24.0 (3.5)	23.4 (3.3)	23.6 (3.2)	0.19
HOMA-IR	2.57 (1.67)	2.34 (1.65)	2.29 (1.63)	0.064
Fasting glucose, mmol/L	5.4 (0.9)	5.4 (0.9)	5.5 (1.0)	0.52
SBP, mmHg	132.1 (18.4)	130.3 (18.3)	131.7 (17.2)	0.72
DBP, mmHg	73.6 (10.0)	73.2 (9.8)	74.5 (10.8)	0.40
Total cholesterol, mmol/L	4.9 (0.9)	4.9 (1.0)	5.0 (1.0)	0.56
HDL, mmol/L	1.3 (0.3)	1.4 (0.3)	1.4 (0.3)	0.020
LDL, mmol/L	3.1 (0.8)	3.0 (0.8)	3.1 (0.9)	0.93
Triglycerides, mmol/L	1.4 (0.7)	1.3 (0.8)	1.5 (0.9)	0.97
*Insulin and IGF axis biomarker*				
Insulin, uIU/mL	10.3 (5.70)	9.4 (5.3)	9.1 (5.3)	0.014
C-peptide, ug/L	2.35 (0.83)	2.15 (0.78)	2.19 (0.87)	0.022
IGF1, ng/mL	152.4 (59.0)	162.3 (62.3)	164.3 (63.8)	0.033
IGFBP3, ug/mL	3.85 (0.92)	3.98 (0.97)	3.89 (0.80)	0.54
IGF1/IGFBP3 ratio, ×10^-3^	39.2 (11.9)	40.6 (12.1)	40.5 (13.1)	0.30
*Clinical characteristic*				
Histological grade, n (%)				
I	57 (26.2)	72 (14.7)	15 (7.5)	<0.001
II	136 (62.4)	336 (68.4)	108 (54.0)	
III	25 (11.5)	83 (16.9)	77 (38.5)	
TNM stage, n (%)				
I	158 (64.0)	327 (59.0)	121 (58.2)	0.49
II	87 (35.2)	225 (40.6)	85 (40.9)	
III	2 (0.8)	2 (0.4)	2 (1.0)
Tumor size, n (%)				
≤2 cm	175 (69.4)	369 (64.5)	136 (63.6)	0.31
>2 cm	77 (30.6)	203 (35.5)	78 (36.5)	
Lymph node status, n (%)				
Negative	220 (87.3)	458 (80.1)	175 (81.8)	0.043
Positive	32 (12.7)	114 (19.9)	39 (18.2)
ER%, n (%)				
Low (<50%)	2 (0.8)	10 (1.8)	20 (9.4)	<0.001
High (≥50%)	250 (99.2)	562 (98.2)	194 (90.7)	
PR status, n (%)				
Negative	12 (4.8)	66 (11.5)	59 (27.6)	<0.001
Positive	240 (95.2)	506 (88.5)	155 (72.4)	
Ki-67 level, n (%)				
Low (<14)	163 (64.7)	302 (52.8)	70 (32.7)	<0.001
High (≥14)	89 (35.3)	270 (47.2)	144 (67.3)	
Molecular subtype, n (%)				
Luminal A	136 (54.0)	188 (32.9)	27 (12.6)	<0.001
Luminal B	116 (46.0)	384 (67.1)	187 (87.4)	
*Adjuvant therapy*				
Chemotherapy, n (%)	39 (15.5)	338 (59.1)	175 (81.8)	<0.001
Radiotherapy, n (%)	98 (38.9)	288 (50.4)	121 (56.5)	<0.001
Endocrine therapy, n (%)	245 (97.2)	557 (97.4)	210 (98.1)	0.79

a Data are mean (SD) for continuous variables or number (%) for categorical variables.

b The mean values (SDs) of the 21-gene RS were 12.6 (4.3), 24.4 (3.7), and 41.0 (13.2) for the low, intermediate, and high categories, respectively.

Percentages may not add up to 100% due to rounding. P values were calculated by general linear model for continuous variables or Chi-square test for categorical variables. The number of missing values was 312 for IGFBP3, 312 for IGF1/IGFBP3 ratio, 129 for histological grade, and 29 for TNM stage.

### Associations of the ΔCt Value for Cancer-Related Genes With Metabolic Factors and Insulin and the IGF Axis Biomarkers

**Figure 1:**
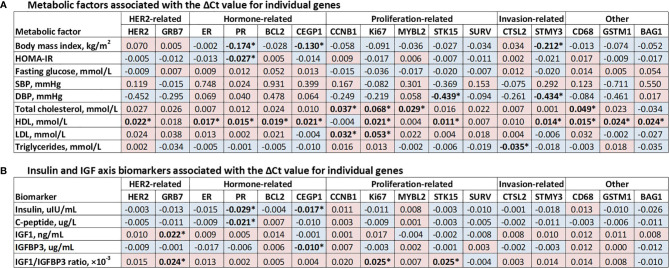
Associations of the ΔCt value for the 16 cancer-related genes with metabolic factors and insulin and the IGF axis biomarkers. **(A)** Associations of the ΔCt value for the 16 cancer-related genes with metabolic factors; **(B)** Associations of the ΔCt value for the 16 cancer-related genes with insulin and IGF axis biomarkers. Data are β estimates with adjustment for age and postmenopausal status (yes, no). Blue cells represent inverse associations and pink cells positive associations, with a statistical significant association marked by asterisk (*). Concentrations of HOMA-IR, IGF1, IGFBP3, IGF1/IGFBP3 ratio, insulin, and C-peptide were log transformed to improve the normality of their distributions.

The ΔCt values of 11 cancer-related genes (HER2, ER, PR, BCL2, CEGP1, Ki67, STK15, STMY3, CD68, GSTM1, and BAG1) were positively associated with HDL (**Figure 1A**). The ΔCt values of several specific cancer-related genes also showed significant associations with BMI (inversely related to PR, CEGP1, and STMY3), HOMA-IR (inversely related to PR), DBP (inversely related to STK15 and STMY3), total cholesterol (positively related to CCNB1, Ki67, MYBL2, and CD68), LDL (positively related to CCNB1 and Ki67), and triglyceride (inversely related to CTSL2).

As for biomarkers of insulin and the IGF axis, the ΔCt values of several hormone-related genes showed inverse associations with insulin (related to PR and CEGP1), C-peptide (related to PR), and IGFBP3 (related to CEGP1) (**Figure 1B**). While the ΔCt values of several HER2-related and proliferation-related genes showed positive associations with IGF1 (related to GRB7) and IGF1/IGFBP3 ratio (related to GRB7, Ki67, and STK15).

### Associations of the 21-Gene RS With Metabolic Factors and Insulin and the IGF Axis Biomarkers

**Figure 2:**
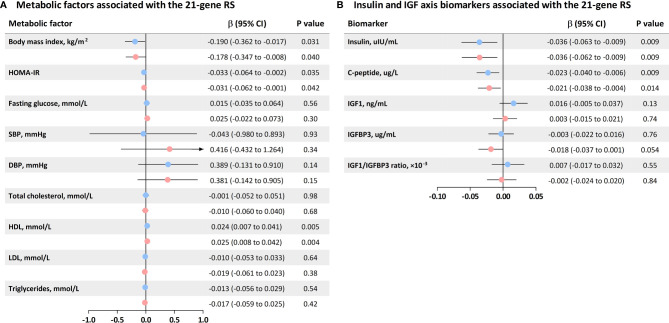
Associations of the 21-gene RS with metabolic factors and insulin and the IGF axis biomarkers. **(A)** Levels of metabolic factors associated with each 10-unit increase in the 21-gene RS; **(B)** Concentrations of insulin and IGF axis biomarkers associated with each 10-unit increase in the 21-gene RS. Blue squares represent unadjusted β estimates, and pink squares indicate β estimates with adjustment for age and postmenopausal status (yes, no). Concentrations of HOMA-IR, IGF1, IGFBP3, IGF1/IGFBP3 ratio, insulin, and C-peptide were log transformed to improve the normality of their distributions.

The 21-gene RS was inversely associated with BMI (β: −0.178 kg/m^2^; 95% CI: −0.347 to −0.008 kg/m^2^) and HOMA-IR (β: −0.031; 95% CI: −0.347 to −0.008) and was positively associated with HDL (β: 0.025 mmol/L; 95% CI: 0.008 to 0.042 mmol/L) after adjustment for age and postmenopausal status (**Figure 2A**).

Regarding biomarkers of insulin and the IGF axis, the 21-gene RS was inversely associated with insulin (β: −0.036 uIU/ml; 95% CI: −0.062 to −0.009 uIU/ml) and C-peptide (β: −0.021 ug/L; 95% CI: −0.038 to −0.004 ug/L) after adjustment for age and postmenopausal status (**Figure 2B**).

### Associations Between the 21-Gene RS and Recurrence by Stratifications of Metabolic Factors and Insulin and the IGF Axis Biomarkers

During a mean follow-up of 36.6 months (SD: 14.4 months), 36 participants with breast cancer recurrence were documented. The cases/1,000 person-years of recurrence was 8.01 for low RS category, 9.19 for intermediate RS category, and 21.00 for high 21-gene RS category (**Supplementary Table 2**). Compared with the low RS category, the high RS category was associated with a greater risk of recurrence (HR: 2.80; 95% CI: 1.08–7.25) with adjustment for age and BMI.

Multivariable-adjusted HR (95% CI) for breast cancer recurrence associated with each 10-unit increase in the 21-gene RS was 1.28 (1.04–1.57) in the overall participants (**Table 2**). When stratified by metabolic risk factors, the risk for recurrence associated with each 10-unit increase in the 21-gene RS was more prominent in participants without obesity (HR: 1.34; 95% CI: 1.08–1.66), without insulin resistance (HR: 1.31; 95% CI: 1.05–1.65), without hyperglycemia (HR: 1.44; 95% CI: 1.17–1.77), without hypertension (HR: 1.28; 95% CI: 1.01–1.63), and without dyslipidemia (HR: 1.29; 95% CI: 1.04–1.60) with full adjustment. No statistically significant interactions between the RS and these metabolic risk factors on breast cancer recurrence were observed (all P for interaction ≥0.15).

**Table 2:** Associations of each 10-unit increase in the 21-gene RS with breast cancer recurrence by metabolic risk factors^a^.

Category	No. of participants	Person-years	Cases	HR (95% CI)^b^	P for interaction
Overall	1038	3096.7	36	1.28 (1.04-1.57)	–
Obesity					
Yes	296	889.4	9	1.50 (0.66-3.45)	0.50
No	742	2207.3	27	1.34 (1.08-1.66)	
Insulin resistance					
Yes	343	1006.5	10	1.32 (0.78-2.24)	0.84
No	695	2090.2	26	1.31 (1.05-1.65)	
Hyperglycemia					
Yes	278	807.7	10	0.74 (0.35-1.54)	0.15
No	760	2289	26	1.44 (1.17-1.77)	
Hypertension					
Yes	350	1058.1	11	1.33 (0.89-1.99)	0.84
No	688	2038.6	25	1.28 (1.01-1.63)	
Dyslipidemia					
Yes	317	954.2	8	1.22 (0.45-3.30)	0.32
No	721	2142.5	28	1.29 (1.04-1.60)	

a Definitions of metabolic risk factors: obesity was defined as BMI ≥25 kg/m^2^; insulin resistance was defined as HOMA-IR ≥2.5; hyperglycemia was defined as fasting glucose ≥5.6 mmol/L; hypertension was defined as systolic blood pressure ≥140 mmHg or diastolic blood pressure ≥90 mmHg; dyslipidemia was defined as total cholesterol ≥6.22 mmol/L, HDL <1.04 mmol/L, LDL ≥4.14 mmol/L, or triglycerides ≥2.26 mmol/L.

b Data were adjusted for age, BMI, postmenopausal status (yes, no), tumor size (≤2 cm, >2 cm), lymph node status (negative, positive), PR status (negative, positive), Ki-67 level (low, high), molecular subtype (Luminal A, Luminal B), chemotherapy (yes, no), radiotherapy (yes, no), and endocrine therapy (yes, no).

When stratified by levels of insulin and the IGF axis biomarkers, participants with low insulin (HR: 1.42; 95% CI: 1.11–1.82), low C-peptide (HR: 1.41; 95% CI: 1.10–1.80), and low IGF1/IGFBP3 ratio (HR: 2.55; 95% CI: 1.18–5.49) exhibited increased risk of breast cancer recurrence (**Table 3**). There were no statistically significant interactions between the 21-gene RS and biomarker levels on the risk of recurrence (all P for interaction ≥0.11).

**Table 3:** Associations of each 10-unit increase in the 21-gene RS with breast cancer recurrence by insulin and the IGF axis biomarker levels^a^.

Category	No. of participants	Person-years	Cases	HR (95% CI)^b^	P for interaction
Overall	1038	3096.7	36	1.28 (1.04-1.57)	–
Insulin					
Low	519	1581.6	20	1.42 (1.11-1.82)	0.22
High	519	1515.1	16	1.12 (0.68-1.84)	
C-peptide					
Low	519	1549.3	18	1.41 (1.10-1.80)	0.47
High	519	1547.4	18	1.12 (0.75-1.67)	
IGF1					
Low	520	1573.8	17	1.27 (0.87-1.87)	0.34
High	518	1522.9	19	1.29 (0.99-1.68)	
IGFBP3					
Low	363	842.8	9	1.62 (0.70-3.75)	0.11
High	363	891.8	10	1.94 (0.95-3.98)	
IGF1/IGFBP3 ratio					
Low	363	860.5	10	2.55 (1.18-5.49)	0.66
High	363	874.1	9	1.63 (0.63-4.27)	

a The number of missing values was 312 for IGFBP3 and 312 for IGF1/IGFBP3 ratio.

b Data were adjusted for age, BMI, postmenopausal status (yes, no), tumor size (≤2 cm, >2 cm), lymph node status (negative, positive), PR status (negative, positive), Ki-67 level (low, high), molecular subtype (Luminal A, Luminal B), chemotherapy (yes, no), radiotherapy (yes, no), and endocrine therapy (yes, no).

## Discussion

This prospective study of 1,038 Chinese women with HR-positive, HER2-negative early-stage breast cancer provided novel insights into the comprehensive relations of the 21-gene RS and the ΔCt values of cancer-related genes with metabolic factors and biomarkers of insulin and the IGF axis, and the prognostic impact of the 21-gene RS in subgroups stratified by the metabolic factors and insulin and the IGF axis biomarkers. The 21-gene RS was inversely associated with BMI, HOMA-IR, insulin, and C-peptide and was positively associated with HDL, which might be driven by the relations between specific cancer-related genes and these metabolic profiles. The 21-gene RS was strongly associated with a higher risk of breast cancer recurrence (28% higher risk per 10-unit increase in the RS), and such prognostic impact was more pronounced among women with relatively favorable metabolic profiles in relevant to an absence of obesity, insulin resistance, hyperglycemia, hypertension, or dyslipidemia (28–44% higher risk per 10-unit increase in the RS) and among women with a low level of insulin, C-peptide, or the IGF1/IGFBP3 ratio (41–155% higher risk per 10-unit increase in the RS).

One noteworthy finding of this study was the significant association patterns of the 21-gene RS and the individual cancer-related genes with specific metabolic factors and biomarkers of insulin and the IGF1 axis. Our results were partly in line with recent studies that revealed an inverse relationship between BMI and the RS in postmenopausal women with hormone receptor-positive, HER2-negative breast cancer (23), and a higher proportion of RS <11 in obese patients compared with non-overweight breast cancer patients (24). Moreover, our findings extended previous evidence by illuminating consistent associations between the 21-gene RS and favorable metabolic profiles, including lower levels of BMI, HOMA-IR, insulin, and C-peptide, and higher levels of HDL. Furthermore, our study provided a detailed whole picture on the associations between the metabolic profiles and specific cancer-related genes, which could explain, at least partially, these observed association patterns. Our observations may reflect potential pathophysiological mechanisms of breast cancer recurrence, which merit further investigations.

Convincing evidence has supported the clinical utility of the 21-gene RS assay as both a prognostic and predictive tool in ER-positive, HER2-negative early-stage breast cancer, independent of clinical characteristics (25–27). More importantly, the prognosis of breast cancer is highly affected by metabolic environment. A series of metabolic risk factors such as obesity, insulin resistance, hyperglycemia, dyslipidemia, and high blood pressure, individually and in combination, could promote adverse breast cancer prognosis through exacerbating adipose inflammation and altering tumor microenvironment (28, 29). Therefore, characterizing the prognostic effect of the 21-gene RS assay among different metabolic subgroups may capture more metabolism-related tumor microenvironment signals and optimize the precision and efficiency of the prediction value of the 21-gene RS assay. Recently, a prospective study of 533 women with stage I-II, hormone receptor-positive, HER2-negative breast cancer showed no significant associations between the 21-gene RS and overall metabolic syndrome status (defined as presence of ≥3 of the following: body mass index ≥27.7 kg/m^2^; hypertension; impaired fasting glucose; HDL <50 mg/dl; hypertriglyceridemia) or any individual components (30). Different from this previous study, our large prospective sample enabled us to assess the relationship between the 21-gene RS and breast cancer recurrence according to multiple metabolic stratifications. Our findings confirmed the strong predictive value of the 21-gene RS assay on breast cancer recurrence and further suggested a more pronounced prognostic impact of the RS assay in breast cancer patients with relatively favorable metabolic profiles, although non-significant interactions indicated no evidence of the effect modification of these metabolic profiles on the relation of the 21-gene RS and breast cancer with breast cancer recurrence.

To the best of our knowledge, this is the first study to elaborate the potential interplay between the 21-gene RS and comprehensive metabolic profiles including common metabolic disorders and insulin and the IGF axis biomarkers in relation to the risk of breast cancer recurrence. The strengths of this study included the relatively large prospective sample, the comprehensive measurements of metabolic profiles, and the well-validated breast cancer prognosis events. First, the relatively short follow-up period and low number of events may limit the power of detecting statistically significant interactions between the 21-gene RS and metabolic stratifications on breast cancer recurrence. Second, although we have carefully controlled for a wide broad of confounders, residual and unmeasured confounding and reverse causality may exist. Third, in this study, the blood samples and baseline clinical measurements were collected from each patient only before the surgical procedure, which may not fully capture the subtle dynamic changes of these values during the operation.

## Conclusions

In this large prospective study, the 21-gene RS was related to favorable metabolic profiles including lower levels of BMI, HOMA-IR, insulin, and C-peptide and higher levels of HDL. Consistently, the prognostic impact of the 21-gene RS was more prominent among Chinese breast cancer patients with relatively favorable metabolic profiles featured by an absence of obesity, insulin resistance, hyperglycemia, hypertension, or dyslipidemia. Our findings highlight the importance of integrating metabolic profiles to optimize the prognostic value of the 21-gene RS.

## Data Availability Statement

The original contributions presented in the study are included in the article/**Supplementary Material**. Further inquiries can be directed to the corresponding authors.

## Ethics Statement

The studies involving human participants were reviewed and approved by Medical Ethics Committee of Ruijin Hospital, Shanghai Jiao Tong University. The patients/participants provided their written informed consent to participate in this study. Written informed consent was obtained from the individual(s) for the publication of any potentially identifiable images or data included in this article.

## Author Contributions

Study concepts: YZ, TW, XC, and KS. Study design: YZ, TW, XC, and KS. Data acquisition: YZ, YT, XC, and KS. Quality control of data and algorithms: YZ, TW, XC, and KS. Data analysis and interpretation: YZ and TW. Statistical analysis: YZ and TW. All authors contributed to the article and approved the submitted version.

## Funding

This work was funded by the financial support from the National Natural Science Foundation of China (81772797, 82072937); Shanghai Municipal Education Commission—Gaofeng Clinical Medicine Grant Support (20172007, 20171901).

## Conflict of Interest

The authors declare that the research was conducted in the absence of any commercial or financial relationships that could be construed as a potential conflict of interest.

## Publisher’s Note

All claims expressed in this article are solely those of the authors and do not necessarily represent those of their affiliated organizations, or those of the publisher, the editors and the reviewers. Any product that may be evaluated in this article, or claim that may be made by its manufacturer, is not guaranteed or endorsed by the publisher.
