# Supplementary material for: 21-Gene Recurrence Assay Associated With Favorable Metabolic Profiles in HR-Positive, HER2-Negative Early-Stage Breast Cancer Patients
Source: Front Endocrinol (Lausanne). 2021 Aug 11;12:725161. doi: 10.3389/fendo.2021.725161 (PMC8385488; doi:10.3389/fendo.2021.725161)
Supplement: Supplementary file 1 [file Table_1.pdf]

Supplementary Material

Table of contents

**Supplementary Table 1.** Characteristics of 21 individual genes	………………………	2
**Supplementary Table 2.** Association between the 21-gene RS and breast cancer recurrence	………………………	3

Supplementary Table 1. Characteristics of 21 individual genes [a]

Gene	Mean (SD)	
	**Ct value**	**ΔCt value**
HER2-related		
HER2	31.58 (1.72)	2.40 (1.30)
GRB7	33.03 (1.30)	3.84 (1.02)
Hormone-related		
ER	29.91 (1.61)	0.72 (1.37)
PR	31.53 (2.19)	2.34 (2.16)
BCL2	31.56 (1.35)	2.37 (1.04)
CEGP1	32.21 (1.99)	3.02 (1.88)
Proliferation-related		
CCNB1	32.57 (1.98)	3.39 (1.91)
Ki67	34.07 (1.38)	4.89 (1.02)
MYBL2	34.78 (2.19)	5.60 (2.26)
STK15	32.82 (2.01)	3.63 (1.77)
SURV	35.13 (1.84)	5.94 (1.90)
Invasion-related		
CTSL2	34.29 (1.64)	5.10 (1.64)
STMY3	30.59 (1.84)	1.41 (1.42)
Other		
CD68	30.82 (1.86)	1.64 (1.49)
GSTM1	32.88 (1.86)	3.69 (1.32)
BAG1	31.48 (1.31)	2.30 (0.97)
Reference gene		
ACTIN	24.93 (1.57)	NA
GAPDH	28.06 (1.42)	NA
GUS	31.95 (1.86)	NA
RPLPO	29.75 (1.79)	NA
TFRC	31.23 (1.56)	NA

[a] There are 16 cancer related genes and 5 reference genes in the 21-gene assay.

Supplementary Table 2. Association between the 21-gene RS and breast cancer recurrence

21-gene RS category	Person-year	Case	Case/1000 person-year	HR (95% CI) [a]	
				Model 1	Model 2
Low (RS <18)	748.6	6	8.01	1.00 (Ref.)	1.00 (Ref.)
Intermediate (RS 18-30)	1632.9	15	9.19	1.08 (0.42-2.81)	1.14 (0.41-3.18)
High (RS ≥31)	714.2	15	21.00	2.80 (1.08-7.25)	2.67 (0.87-8.17)

The number of participants was 252 in low RS category, 572 in intermediate RS category, and 214 in high RS category.

[a] Model 1 was adjusted for age and BMI. Model 2 was adjusted for age, BMI, postmenopausal status (yes, no), tumor size (≤2 cm, >2 cm), lymph node status (negative, positive), PR status (negative, positive), Ki-67 level (low, high), molecular subtype (Luminal A, Luminal B), chemotherapy (yes, no), radiotherapy (yes, no), and endocrine therapy (yes, no).
